# Can telemedicine improve adherence to medical treatment? Lessons learned from research on acromegaly conducted during COVID-19 pandemic

**DOI:** 10.20945/2359-3997000000576

**Published:** 2022-11-28

**Authors:** Cesar Luiz Boguszewski

**Affiliations:** 1 Serviço de Endocrinologia e Metabologia do Hospital de Clínicas da Universidade Federal do Paraná Departamento de Clínica Médica Curitiba PR Brasil Serviço de Endocrinologia e Metabologia do Hospital de Clínicas da Universidade Federal do Paraná (SEMPR), Departamento de Clínica Médica, Curitiba, PR, Brasil

At the time I write this Editorial, most of the world is transitioning out of the acute COVID-19 phase thanks to unprecedented health-related innovations that allowed development of several COVID-19 tests, clinical trials and vaccines in just few months after the outbreak of the disease. Nevertheless, the COVID-19 pandemic was undoubtedly one of the greatest health challenges ever faced by the international community and presented many indirect impacts on the public health systems, particularly for vulnerable populations (1).

One of the main impacts observed in the Brazilian public health system was the promotion of regular use of telemedicine, a tool that has transformed the provision of medical services during COVID-19 pandemic (2). In this issue of the *Archives of Endocrinology and Metabolism*, Jesus Nunes and cols. (3) reported their experience with telemedicine in the management of patients with acromegaly followed in an academic reference center in the Brazilian state of Ceara during the first wave of SARS-CoV-2 infection in 2020. Theoretically, acromegaly could increase the risk of contracting COVID-19 because its frequent association with diabetes, hypertension, obstructive sleep apnea, macroglossia, and respiratory disfunction caused by the presence of multiple anatomical abnormalities (4). On the other hand, biochemical control has been shown to reverse or improve morbimortality of the disease (5). In a substantial number of patients, continuous medical therapy with somatostatin receptor ligands (SRL) is necessary to obtain adequate disease control, a costly treatment that requires the patient to have special prescription and documentation to pick up the medication at a specialized pharmacy, and a specialized nurse or care provider for administration of the monthly injections. Taken together, these factors explain the concerns about the adherence of acromegaly patients to their visits and medical treatment during the COVID-19 pandemic.

In their trial, Jesus Nunes and cols. (3) applied a standard questionnaire to 101 acromegaly patients (mean age of 56 years) through telemedicine, revealing that 19 subjects discontinued treatment during the pandemic for fear of leaving home, and 17 of them were receiving SRL. Additionally, they identified other 8 subjects who missed follow-up for more than one year due to other reasons unrelated to the pandemic. However, the authors' conclusion that the pandemic had a significant impact in the treatment of their patients is somewhat misleading, given that excluding those 8 patients who missed follow-up for reasons unrelated to the COVID-19, we had seventy-four (80%) subjects of the interviewed group that did not miss follow-up and received their injections regularly. Unfortunately, we lack information on how many individuals in this group were on medical therapy with SRL to compare with those 17 who discontinued treatment. This comparison would be important to prove that the fear of contracting SARS-CoV-2 infection was, indeed, the main reason for the worse adherence and to identify potential differences between adherent and non-adherent subjects. Nevertheless, the most striking contribution of their study was the demonstration that 24 (almost 90%!) of the interviewed acromegaly patients who missed follow-up resumed it after being contacted. This finding indicates that telemedicine plays an important role that can be useful not only during periods of pandemics, but also during regular times to improve therapeutic adherence in chronic diseases. Moreover, telemedicine might be a tool to unburden the outpatient visits in the health system which, in turn, might prove to be a cost-effective approach to be regulated and implemented.
